# Capecitabine metronomic chemotherapy for metastatic colorectal cancer patients reaching NED: A protocol for a prospective, randomized, controlled trial

**DOI:** 10.1371/journal.pone.0320591

**Published:** 2025-04-21

**Authors:** Jiaming Wu, Yu Dong, Wanshan Zhu, Jincheng Meng, Huatang Zhang, Cantu Fang, Lizhu Lin

**Affiliations:** 1 Zhongshan Hospital of Traditional Chinese Medicine Affiliated to Guangzhou University of Chinese Medicine, Zhongshan, China; 2 The First Clinical College of Guangzhou University of Chinese Medicine, Guangzhou, China; 3 Clinical Research and Big Data Laboratory, South China Research Center for Acupuncture and Moxibustion, Medical College of Acu-Moxi and Rehabilitation, Guangzhou University of Chinese Medicine, Guangzhou, China; Peter MacCallum Cancer Institute, AUSTRALIA

## Abstract

**Introduction:**

An increasing number of patients with metastatic colorectal cancer (mCRC) have achieved no evidence of diseases (NED) status after surgery or other treatments. However, the latest guidelines for colorectal cancer do not recommend an appropriate treatment for patients with mCRC who achieve NED status. Capecitabine metronomic chemotherapy has the advantages of significant efficacy and minimal adverse reactions, it is a potential effective method for maintenance treatment for mCRC, but no RCTs have been reported. Therefore, we designed a randomized controlled trial to evaluate the efficacy and safety of capecitabine metronomic chemotherapy for mCRC patients who achieve NED.

**Methods/design:**

This study is a prospective, randomized controlled study that evaluates the efficacy and safety of capecitabine metronomic chemotherapy for patients with mCRC who achieve NED status. 240 eligible participants will be randomly assigned to either a capecitabine metronomic chemotherapy group or a “watch and wait” group at a 1:1 allocation ratio. Eligible patients diagnosed with stage IV mCRC, both the primary tumor and the metastases, are those who have achieved R0 resection (or complete destruction by ablation) and reached NED. Participants who are enrolled in the capecitabine group will receive capecitabine (500 mg/m^2^ body surface area twice daily) for 2 years. Meanwhile, those who are assigned to the control group will receive regular imaging examination and follow-up only. All participants will follow up for 1 year after receiving 2 years of intervention. The primary outcomes will be disease-free survival (DFS) from randomization, stratified by preoperative chemotherapy, metastatic organs, number of metastases, lenght of previous systemic treatment, response to previous chemotherapy. Secondary outcomes will include overall survival (OS), 1-year,2-year,3-year survival rate and adverse reactions.

**Discussion:**

As a potentially effective treatment, low-dose capecitabine metronomic chemotherapy has been explored in clinical practice. The results of this trial will provide evidence on the efficacy and safety of capecitabine metronomic chemotherapy for patients with mCRC who have reached NED status.

**Trial registration:**

Chinese Clinical Trial Registry (ChiCTR2100047149, protocol version number F2.0)

## Introduction

Metastatic colorectal cancer (mCRC) accounts for more than 40% of the total colorectal cancer population [1,2]. In recent years, with the progress of surgery and a series of drugs to improve the objective response rate of tumors, more and more patients with mCRC can now receive surgical resection after conversion therapy, or achieving no evidence of disease (NED) status through local treatment such as ablation [3,4]. This has greatly improved survival for patients with mCRC. At present, a total of 6 months of perioperative chemotherapy is routinely recommended for patients with mCRC after resection, but many patients have postoperative recurrence and metastasis. For patients with mCRC who achieve NED status after resection, how to avoid tumor recurrence and metastasis after surgery is an urgent clinical problem [5,6].

Approximately 60–70% of patients diagnosed with stage IV colorectal cancer will experience tumor recurrence and metastasis following surgical intervention. Previous studies(JCOG0603, EORTC40983) have demonstrated that adjuvant chemotherapy following surgical intervention for metastatic colorectal cancer is associated with a prolonged duration of disease-free survival (DFS); however, no significant enhancement in overall survival has been observed [7,8]. The presence of Minimal Residual Disease (MRD) or Circulating Tumor Cell (CTC) significantly contributes to the postoperative recurrence of colorectal cancer. The efficacy of adjuvant chemotherapy may be compromised by drug resistance and the immunosuppressive microenvironment within the human body, necessitating the development of novel therapeutic approaches.

Fluorouracil (5 - FU) is the most important and basic drug used in chemotherapy for colorectal cancer. Capecitabine is an oral drug and a member of the fluorouracil class. The highly expressed thymidine phosphorylase in tumor tissue can degrade it into 5-FU, significantly increasing the drug concentration at the tumor site, and simulating continuous perfusion of 5-FU, so that it maintains a stable blood drug concentration at the action site [9]. Single-agent capecitabine is commonly administered to mCRC patients who exhibit intolerance towards multi-drug combination chemotherapy. However, the conventional dosage (Capecitabine 1250mg/m^2^ body surface area twice daily) poses challenges such as significant toxic reactions, immunosuppression, and drug resistance [10,11].

Capecitabine metronomic chemotherapy is a mode of small dose, continuous administration and oral administration which can maintain drug concentration at a low and effective blood drug concentration for a long time, prolong the disease control time, and mitigate the toxic side effects. Metronomic chemotherapy exerts an anti-tumor effect by inhibiting tumor angiogenesis, and can promote and induce anti-tumor immune response in the microenvironment. Its targets are vascular endothelium and tumor stromal cells, tumor stem cells and immune cells, but not the tumor itself. It can shorten the treatment interval, reduce the recovery time for tumor cells during the treatment interval, and inhibit tumor proliferation by anti-angiogenesis, immune activation, reducing drug resistance and inducing tumor dormancy to inhibit tumor proliferation [12–16].

After multiple courses of combined chemotherapy for advanced colorectal cancer, patients’ sensitivity to drugs will decline, and the adverse reactions of the chemotherapy drugs will continue to accumulate. It is necessary to balance the anti-tumor effect while reducing the treatment-related adverse reactions. Capecitabine metronomic chemotherapy has shown good efficacy in breast cancer and head and neck cancer [17]. The SYSUC-001 study [18] analyzed the efficacy of capecitabine metronomic chemotherapy after standard treatment in patients with triple-negative breast cancer. 434 patients were randomized to either a capecitabine metronomic chemotherapy group (650 mg/m^2^ twice daily for 1 year) or an observation group. The findings showed that 5-year DFS significantly improved in the capecitabine metronomic chemotherapy group versus the observation group (83% vs. 73%). A multicentre, randomised, controlled, phase 3 trial have shown that capecitabine metronomic chemotherapy improved failure-free survival in patients with high-risk locoregionally advanced nasopharyngeal carcinoma [19]. However, there is currently a lack of prospective randomized controlled trials (RCTs) assessing the efficacy and safety of capecitabine in patients with mCRC following achieving NED status. The purpose of this trial is to evaluate the effect of capecitabine metronomic chemotherapy for mCRC patients reaching NED state.

## Methods and analysis

### Study design

**Fig 1:**
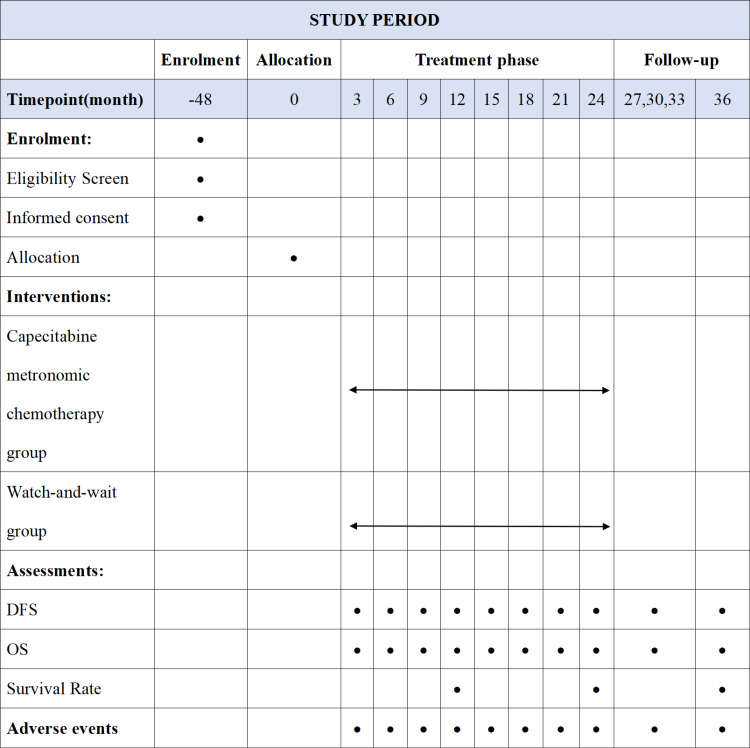
Timeline and schedule of enrolment, interventions and assessments.

This study is a prospective, randomized controlled study of capecitabine metronomic chemotherapy for mCRC patients reaching NED. All eligible individuals (240) will be randomly assigned to either a capecitabine metronomic chemotherapy group (*n* = 120) or a “watch-and-wait” group (*n* = 120). This study protocol has been approved by the Ethics Committee at Zhongshan Hospital of Traditional Chinese Medicine Affiliated to Guangzhou University of Chinese Medicine (No. 2021ZSZY-LLK-354) and registered in the Chinese Clinical Trial Registry (ChiCTR2100047149). All eligible participants will be required to sign an informed consent form prior to random grouping. The overall timeline and study procedures are outlined in Fig 1.

### Study subjects

All subjects will be recruited through posters at Zhongshan Hospital of Traditional Chinese Medicine Affiliated to Guangzhou University of Chinese Medicine.It will be conducted from December 2021 to December 2025.All subjects must be medically fit patients with mCRC advanced colon cancer who had achieved NED status after surgery or ablation, aged 18–80 years, and with an ECOG score of 0–1, and who have completed perioperative chemotherapy. Major exclusion criteria are multiple primary carcinoma patients; pregnant women; mentally ill patients; patients with other serious underlying diseases. Complete inclusion and exclusion criteria are outlined in Table 1.

**Table 1:** Study inclusion and exclusion criteria.

Inclusion criteria	Exclusion criteria
(1) Male or female aged 18–80 years.	(1) Patients diagnosed with more than one malignancy.
(2) Pathologically confirmed metastatic colorectal adenocarcinoma.	(2) Pregnant or lactating women.
(3) Contrast-enhanced CT or MRI of the chest and abdomen at the initial diagnosis, clinical stage IV colon cancer with liver and/or lung metastasis, and potentially curative R0 resection (including local therapy such as radiofrequency ablation) for the primary tumor and metastatic lesions, have completed perioperative chemotherapy, and achieved NED status on chest and abdomen CT or MRI within 4 weeks before enrollment.	(3) Patients with mental illness.
(4) ECOG score of 0–1.	(4) Combined with coronary heart disease, myocardial infarction, renal failure, pulmonary embolism and other diseases that may affect the survival of patients.
(5) There were no other underlying diseases affecting organ function.	(5) Patients with poor compliance can not cooperate with the treatment.
(6) Understand, be willing to give consent, and sign the written ICF prior to undergoing any study-specific procedure.	(6) There is a known deficiency of DPD, or Capecitabine intolerance.

Abbreviation: NED, no evidence of disease; ECOG, Eastern Co-operative Oncology Group; ICF, informed consent form; DPD, dihydropyrimidine dehydrogenase.

### Randomization, allocation and blinding

**Fig 2:**
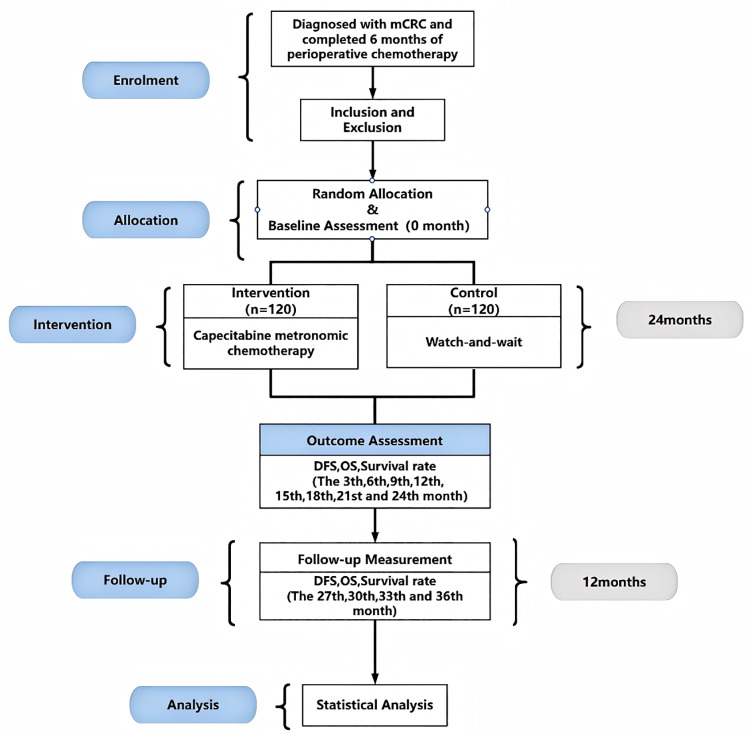
Flowchart of trial procedures.

Eligible patients will be randomly assigned to either a watch-and-wait group or a capecitabine metronomic chemotherapy group in equal proportions via the random number table method. After the participants sign the informed consent form and complete the baseline assessment, the investigators will assign them a random number and assign the participants to the appropriate group. Participants will be informed that they have an equal chance of being assigned to the capecitabine metronomic chemotherapy group or to the watch-and-wait group. Subject assignment will be performed by an independent investigator in the clinical department who will not be involved in the evaluation of the results. The outcome assessors, data collectors, and statisticians will be blinded to the intervention assignment, but the patients participating in the study will not be blinded. Single blinding of the outcome assessors and data analysts will be maintained throughout the course of the study.The overall study design is illustrated in Fig 2.

### Intervention

The interventions studied will be implemented by attending physicians with at least 3 years of clinical experience in the Oncology Department at Zhongshan Affiliated Hospital, Guangzhou University of Chinese Medicine. Based on chemotherapy theory for metastatic colorectal cancer and previous randomized controlled trials, capecitabine chemotherapy process interventions will be developed under the consensus of colorectal cancer oncology experts. Prior to initiating the study, the clinical practitioner will receive specialized training to gain a comprehensive understanding of capecitabine metronomic chemotherapy, and to administer treatment in accordance with the specific requirements of the investigator’s manual. All eligible patients in both groups will have been diagnosed with stage IV colon cancer with hepatic and/or lung metastases, have potentially cured R0 resection of the primary tumor and metastatic lesion (including local treatments such as radiofrequency ablation), have completed perioperative chemotherapy, and have reached NED status on computed tomography (CT) or MRI within 4 weeks of enrollment. The study will be conducted over a 3-year period, with all treatments completed within the first 2 years. Follow-up will last for 12 months, beginning after the completion of the 24-month intervention period.

#### Capecitabine metronomic chemotherapy group.

All eligible patients will have been diagnosed with Stage IV mCRC according to the National Cancer Network (NCCN) colorectal cancer diagnosis and treatment guidelines. They also will have achieved NED after local treatment such as surgery or ablation and completed perioperative 6-month chemotherapy.

In the capecitabine metronomic chemotherapy group, after chemotherapy ends, patients will continue to take capecitabine (500 mg/m^2^ bid, uninterrupted) for 2 years, or until the disease progresses or the patient develops intolerable side effects. Retrieve the medication box and conduct a count of the remaining quantity to assess adherence, including instances of underdosing, overdosing, or loss of medication. During the trial period, each observation index will be re-visited every 3 months from postoperative intervals to determine whether there is any recurrent metastasis of the tumor. The follow-up will last for 1 year, and the observation indicators will be re-visited every 3 months.

#### Watch-and-wait group.

Eligible patients diagnosed with stage IV mCRC, both the primary tumor and the metastases, have achieved R0 resection (or complete destruction by ablation) and reached NED. After completing 6 months of perioperative chemotherapy, they will enter observational waiting.

The observation period will be 2 years, and the observation indicators will be re-visited every 3 months; the follow-up period will be 1 year. The enrolled patients will be reexamined with enhanced CT or MR every 3 months during the observation period and the follow-up period to determine whether there is any recurrence or metastasis of the tumor.

## Outcome

The primary endpoints disease-free survival (DFS) will be analyzed by using intention-to-treat (ITT) data set from randomization, stratified by preoperative chemotherapy, metastatic organs, number of metastases, lenght of previous systemic treatment (under 6 months, over 6 months), response to previous chemo: CR/PR or SD/PD. Secondary endpoints will include overall survival (OS), 1-year, 2- year and 3-year survival rate. The adverse reactions will be counted, and the bone marrow suppression rate and the incidence of digestive tract reactions will be used as safety evaluations.

### Primary outcomes

#### Disease-free survival (DFS).

Disease-free survival (DFS) refers to the survival time calculated from randomization without any locoregional progressive disease, distant metastasis, recurrence or secondary colorectal cancer. Death from any cause will also be considered an event for this endpoint.

The enrolled patients will be assessed every 3 months starting from the baseline (Month 0). At the same time, the enhanced CT or MR will be reexamined, and combined with the tumor marker level to evaluate the recurrence and metastasis of the tumor, to observe patient survival, and to calculate the DFS.

### Secondary outcome

#### Overall survival (OS).

Another primary outcome is patient overall survival (OS), which is the time from randomization to death from any cause. For patients who are lost to follow-up before death, the time of last follow-up will be calculated as the time of death.

Participating patients will be recorded from baseline (month 0), with visits every 3 months to document patient survival.

#### Survival rate (DFS and OS).

After visits at the time nodes specified in the trial, patient survival will be calculated at the time nodes of the 1^st^ year, 2^st^ year and the 3^nd^ year after randomization.

#### Safety.

All participants will undergo physical tests to evaluate their bodies before screening enrolling and after treatment. These tests will include the function of the heart, liver, kidneys, and other organs, including cardiac ultrasound, ECG, white blood cell count, hematocrit, platelets, aspartate aminotransferase/alanine transaminase, blood urea nitrogen, creatinine, γ-glutamyl transpeptidase, and erythrocyte sedimentation rate.

Adverse events (AEs) are any unexpected adverse conditions, symptoms, or sensations that occur in subjects throughout the trial period, whether or not they are associated with capecitabine treatment. AE will be graded using the Common Terminology Standard (CTCAE) 4.0.3 [20]. A serious adverse event (SAE) will be defined as any of grade 3, 4, or 5 adverse events. Each AE will be recorded on the case report form (CRF). SAE will be reported to the Competent Authorities and the Ethics Committee. Initiators must submit annual safety reports to Competent Authorities and Ethics Committees during clinical trials. At the same time, the incidence of bone marrow suppression and the incidence of gastrointestinal responses will be counted to assess the safety of capecitabine metronomic chemotherapy.

### Quality control, data management and monitoring

Prior to recruitment, research team members will be required to attend a training workshop to ensure their strict adherence to the research protocol, and that they have a comprehensive understanding of the trial management process. The intervention will be implemented by attending physicians with at least 3 years of hospital experience. The trial process will be conducted in accordance with a researcher’s manual of standard operating procedures developed in consultation with experts with extensive therapeutic experience. To improve compliance, we maintained contact with the subjects and at least one family member of the subjects, and regular telephone follow-up was conducted.

This data will be collected and recorded on CRF. All data will be input to a password-protected computer by a staff member who does not know how the group assignments were made. It will be checked twice by investigators after the data is entered. The collected clinical data will be entered into the database and the database will be regularly updated according to follow-up timing. The original CRF and all database data will be securely stored in the Oncology Department building at Zhongshan Hospital of Traditional Chinese Medicine affiliated to Guangzhou University of Chinese Medicine. Throughout the trial, two trained, qualified, and independent supervisors will regularly visit the trial center to be responsible for data monitoring, regulatory management, and to conduct a trial audit every 3 months, independent of the investigator and the organizer. This will include specification of the random inspection program, the reasonableness of the inclusion and exclusion criteria, the proper implementation, the implementation of informed consent procedures, the specification of the CRF, the confidential updating of the database, and SAE reporting, and also determining whether to terminate the study prematurely. For any cases in which there is shedding or loss of visit, it will be necessary to explain the reasons for the shedding and the loss of visit, and calculate the proportion of cases in which there was shedding and loss of visit relative to the total number of observed cases.

### Sample size calculation

The sample size estimation was conducted using the Logrank Test, with disease-free survival (DFS) as the primary outcome measure. According to the JCOG0603 study, patients with mCRC who achieved NED status and received adjuvant chemotherapy had a 3-year DFS rate of 52.1% [7]. Based on the data from the phase II single-arm CAMCO trial [21], in conjunction with clinical expertise and expert opinions, We hypothesized that the 3-year DFS rate in the group receiving capecitabine metronomic chemotherapy would be 67.1%. We will need at least 119 participants in the intervention and control group, 238 participants in total (alpha = 0.05, power=0.90,). A drop-out of 5% is anticipated [22]. The sample size of this study was determined to be 240 participants, taking into account potential additional dropouts.

We calculated the sample size using the Lakatos method, which accounts for varying accrual times, dropout rates, and study length—critical factors for trials with survival endpoints like DFS. While the Freedman method is widely used for its simplicity, it assumes constant hazard ratios and patient compliance, which may not fully reflect the complexities of real-world trials. The Lakatos method [23], though more restrictive in its assumptions, offers a more realistic model for time-dependent variables and survival data, making it a better fit for our single-center study [24].

However, some assumptions of the Lakatos method may not hold in practice, potentially resulting in a wider confidence interval (CI) for the hazard ratio (HR). Although the Lakatos method may yield a smaller sample size compared to Freedman’s, we have carefully balanced statistical rigor with the feasibility of a single-center trial, given our available resources. To mitigate the risk of wide CIs, we will closely monitor accrual patterns and dropout rates throughout the study to ensure robust results.

### Statistical analysis

The statistical analysis will use SPSS version 26.0 (IBM SPSS Statistics, New York, USA). All analysis will be based on the ITT dataset. Missing data will be supplemented with the principle of multiple interpolation. All statistical tests will use a two-side test, and *P* < 0.05 will be considered statistically significant for the difference being tested. The survival analysis will use the Kaplan-Meier method for one-factor survival, calculating the median disease DFS and OS of the two groups, and comparing the survival curves by the Logrank test. The χ² test will be used to analyze the counting data of adverse events (AEs). Additionally, the 95% confidence interval (CI) for the observed hazard ratio (HR) will be calculated at the end of the trial. *P* <0.05 will be considered statistically significant.

## Discussion

In the past, mCRC was generally regarded as an unresectable disease with no chance of cure. Conversion therapy refers to the treatment of initially unresectable mCRC patients, through effective chemotherapy and targeted therapy so that the tumor will retreat to the condition of surgical resection. Several clinical studies have used chemotherapy combined with targeted therapy for patients with initially unresectable metastatic colorectal cancer. In these studies, tumor regression depth, objective response rate (ORR) and radical surgery resection rate have significantly improved, such that more and more patients with mCRC have the opportunity to receive radical surgery [25].

**Fig 3:**
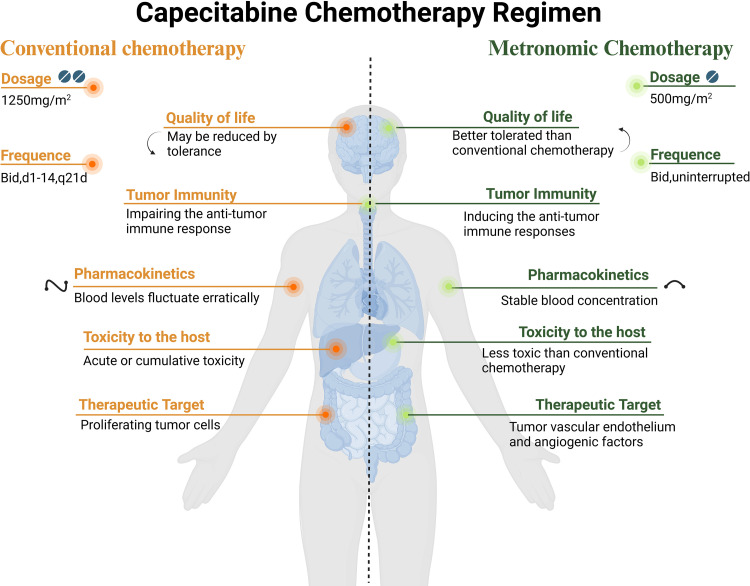
Differences between conventional and metronomic chemotherapy utilizing capecitabine.

For patients with mCRC who achieve NED in the tumor-free state after radical surgery, there are no RCT study data to address whether to continue to use chemotherapy, which treatment regimen to use, or the duration of chemotherapy. Based on expert opinion and research data on postoperative adjuvant chemotherapy for stage III colorectal cancer [26], 6 months of perioperative chemotherapy and watch-and-wait after chemotherapy are generally recommended [27,28]. However, in clinical practice, it has been found that some patients with mCRC still have recurrence and metastasis within a short time after reaching NED. Thus, there is an urgent need for an effective treatment strategy with low toxicity and long-term drug tolerance. Metronomic low dose capecitabine chemotherapy has the advantages of minimal side effects, convenient long-term administration, and a clear anti-tumor effect(see Fig 3 for details). Moreover, the efficacy of metronomic capecitabine chemotherapy has been verified in breast cancer and nasopharyngeal carcinoma. As a potentially effective treatment, low-dose capecitabine metronomic chemotherapy has been explored in clinical practice. The purpose of this proposed clinical study is to verify the treatment efficacy by evidence-based medicine.

## Conclusion

mCRC patients after reaching NED state still lack an effective standard therapy, and thus the proposed study will primarily observe capecitabine metronomic chemotherapy for mCRC after reaching NED state maintenance treatment regarding the DFS, OS, the quality of life score and adverse reaction data. This study is expected to change clinical guidelines, to provide high-quality clinical research data for clinical practice of such patients.

### Trial status

This trial is currently recruiting patients, which started on 31 December 2021 and is anticipated to end on 31 December 2025. The protocol is Version 3.0 dated 2023/01/24.

**Strengths and limitations of this study**

- This is the first prospective, randomized, controlled trial to evaluate the efficacy and safety of capecitabine metronomic chemotherapy for mCRC patients who achieve NED status.
- This study will assess clinically relevant intermediate outcomes, including Disease-free survival (DFS), overall survival, 1-year,2-year,3-year survival rate and adverse reactions.
- This study protocol was developed based on validated evidence, which was acknowledged by oncologists and statisticians, thereby demonstrating its feasibility and scientific rigor.
- The limitations of our study include the single-center design and the relatively small sample size due to constraints in time and resources, which may leave some uncertainty regarding the true value of Capecitabine metronomic chemotherapy.

## Supporting information

S1 File Standard Protocol Items: Recommendations for Interventional Trials (SPIRIT) Checklist. (DOC)

## Abbreviations:

mCRC: metastatic colorectal cancer
NED: no evidence of disease (NED)
CT: computed tomography
PFS: progression-free survival
OS: overall survival
ECOG: Eastern Cancer Cooperation Group
AE: adverse event
SAE: serious adverse event
CRF: case report form
ORR: objective response rate

## References

[1] Sung H, Ferlay J, Siegel R. Global cancer statistics 2020: GLOBOCAN estimates of incidence and mortality worldwide for 36 cancers in 185 countries. CA. 2021;71(3):209–49. doi: 10.3322/caac.21660 33538338

[2] Zheng R, Sun K, Zhang S. Report of cancer epidemiology in China, 2015. Zhonghua Zhong Liu Za Zhi. 2019;41(1):19–28. 10.3760/cma.j.issn.0253-3766.2019.01.005 30678413

[3] Dienstmann R, Mason MJ, Sinicrope FA, Phipps AI, Tejpar S, Nesbakken A, et al. Prediction of overall survival in stage II and III colon cancer beyond TNM system: a retrospective, pooled biomarker study. Ann Oncol. 2017;28(5):1023–31. doi: 10.1093/annonc/mdx052 28453697 PMC5406760

[4] Thirunavukarasu P, Talati C, Munjal S, Attwood K, Edge SB, Francescutti V. Effect of Incorporation of Pretreatment Serum Carcinoembryonic Antigen Levels Into AJCC Staging for Colon Cancer on 5-Year Survival. JAMA Surg. 2015;150(8):747–55. doi: 10.1001/jamasurg.2015.0871 26083632

[5] National Comprehensive Cancer Network Guidelines: Colorectal cancer. Version 2. 2022.

[6] Yoshino T, Argilés G, Oki E, Martinelli E, Taniguchi H, Arnold D, et al. Pan-Asian adapted ESMO Clinical Practice Guidelines for the diagnosis treatment and follow-up of patients with localised colon cancer . Ann Oncol. 2021;32(12):1496–510. doi: 10.1016/j.annonc.2021.08.1752 34411693

[7] Kanemitsu Y, Shimizu Y, Mizusawa J. Hepatectomy followed by mFOLFOX6 versus hepatectomy alone for liver-only metastatic colorectal cancer (JCOG0603): A phase II or III randomized controlled trial. J Clin Oncol. 2021;39(34):3789–99. doi: 10.1200/JCO.2020.38.15_suppl.1234 34520230

[8] Nordlinger B, Sorbye H, Glimelius B, Poston GJ, Schlag PM, Rougier P, et al. Perioperative FOLFOX4 chemotherapy and surgery versus surgery alone for resectable liver metastases from colorectal cancer (EORTC 40983): long-term results of a randomised, controlled, phase 3 trial. Lancet Oncol. 2013;14(12):1208–15. doi: 10.1016/S1470-2045(13)70447-9 24120480

[9] Lam SW, Guchelaar HJ, Boven E. The role of pharmacogenetics in capecitabine efficacy and toxicity. Cancer Treat Rev. 2016;50:9–22. doi: 10.1016/j.ctrv.2016.08.001 27569869

[10] Pouya FD, Rasmi Y, Camci IY, Tutar Y, Nemati M. Performance of capecitabine in novel combination therapies in colorectal cancer. J Chemother. 2021;33(6):375–89. doi: 10.1080/1120009X.2021.1920247 34019782

[11] Wu Z, Deng Y. Capecitabine versus continuous infusion fluorouracil for the treatment of advanced or metastatic colorectal cancer: a meta-analysis. Current Treatment Options in Oncology. 2018;19(12):77. 30483908 10.1007/s11864-018-0597-y

[12] Pasquier E, Kavallaris M, André N. Metronomic chemotherapy: new rationale for new directions. Nat Rev Clin Oncol. 2010;7(8):455–65. doi: 10.1038/nrclinonc.2010.82 20531380

[13] Woo I, Jung Y. Metronomic chemotherapy in metastatic colorectal cancer. Cancer Letters. 2017;400(1):319–24. 28274890 10.1016/j.canlet.2017.02.034

[14] Biziota E, Mavroeidis L, Hatzimichael E. Metronomic chemotherapy: A potent macerator of cancer by inducing angiogenesis suppression and antitumor immune activation. Cancer Letters. 2017;400(1):243–51. 28017892 10.1016/j.canlet.2016.12.018

[15] Bondarenko M, Le Grand M, Shaked Y, Raviv Z, Chapuisat G, Carrère C, et al. Metronomic chemotherapy modulates clonal interactions to prevent drug resistance in non-small cell lung cancer. Cancers (Basel). 2021;13(9):2239. doi: 10.3390/cancers13092239 34066944 PMC8125381

[16] Bocci G, Kerbel RS. Pharmacokinetics of metronomic chemotherapy: a neglected but crucial aspect. Nat Rev Clin Oncol. 2016;13(11):659–73. doi: 10.1038/nrclinonc.2016.64 27184418

[17] Kerbel R, Andre N. Adjuvant metronomic chemotherapy for locoregionally advanced nasopharyngeal carcinoma. Lancet. 2021;398(10297):278–9. 34111417 10.1016/S0140-6736(21)01240-X

[18] Wang X, Wang S-S, Huang H, Cai L, Zhao L, Peng R-J, et al. Effect of Capecitabine maintenance therapy using lower dosage and higher frequency vs observation on disease-free survival among patients with early-stage triple-negative breast cancer who had received standard treatment: the SYSUCC-001 randomized clinical trial. JAMA. 2021;325(1):50–8. doi: 10.1001/jama.2020.23370 33300950 PMC7729589

[19] Chen Y, Liu X, Zhou Q. Metronomic capecitabine as adjuvant therapy in locoregionally advanced nasopharyngeal carcinoma: a multicentre, open-label, parallel-group, randomised, controlled, phase 3 trial. Lancet. 2021;398(10297):303–13. 34111416 10.1016/S0140-6736(21)01123-5

[20] U.S. department of health and human services. National Institutes of Health NCI. Common Terminology Criteria for Adverse Events (CTCAE), v4.03. 2010.

[21] Ling J, Lin Z, Shi L, Lin Y, Liu X, Lin J, et al. Capecitabine maintenance therapy in metastatic colorectal cancer patients with no evidence of disease: CAMCO trial. Future Oncol. 2023;19(30):2045–54. doi: 10.2217/fon-2023-0149 37814832

[22] Teare MD, Dimairo M, Shephard N, Hayman A, Whitehead A, Walters SJ. Sample size requirements to estimate key design parameters from external pilot randomised controlled trials: a simulation study. Trials. 2014;15:264. doi: 10.1186/1745-6215-15-264 24993581 PMC4227298

[23] Lakatos E. Sample sizes based on the log-rank statistic in complex clinical trials. Biometrics. 1988;44(1):229–41. doi: 10.2307/2531910 3358991

[24] Lu K. Sample size calculation for logrank test and prediction of number of events over time. Pharm Stat. 2021;20(2):229–44. doi: 10.1002/pst.2069 32909395

[25] Hu H, Wang K, Huang M, Kang L, Wang W, Wang H, et al. Modified FOLFOXIRI with or without cetuximab as conversion therapy in patients with RAS/BRAF wild-type unresectable liver metastases colorectal cancer: the FOCULM multicenter phase II trial. Oncologist. 2021;26(1):e90–8. doi: 10.1634/theoncologist.2020-0563 33400355 PMC7794191

[26] Tsuji A, et al. The randomized phase II study of FOLFOXIRI plus cetuximab versus FOLFOXIRI plus bevacizumab as the first-line treatment in metastatic colorectal cancer with RAS wild-type tumors: The DEEPER trial (JACCRO CC-13). Proceedings of the American Society of Clinical Oncology (ASCO). 2021:abs3501.

[27] Shah MA, Renfro LA, Allegra CJ, André T, de Gramont A, Schmoll H-J, et al. Impact of Patient Factors on Recurrence Risk and Time Dependency of Oxaliplatin Benefit in Patients With Colon Cancer: Analysis From Modern-Era Adjuvant Studies in the Adjuvant Colon Cancer End Points (ACCENT) Database. J Clin Oncol. 2016;34(8):843–53. doi: 10.1200/JCO.2015.63.0558 26811529 PMC4872008

[28] Auclin E, Zaanan A, Vernerey D, Douard R, Gallois C, Laurent-Puig P, et al. Subgroups and prognostication in stage III colon cancer: future perspectives for adjuvant therapy. Ann Oncol. 2017;28(5):958–68. doi: 10.1093/annonc/mdx030 28453690

